# Supplementary material for: Immunohistochemical Detection of Estrogen Receptor-Beta (ERβ) with PPZ0506 Antibody in Murine Tissue: From Pitfalls to Optimization
Source: Biomedicines. 2022 Dec 1;10(12):3100. doi: 10.3390/biomedicines10123100 (PMC9775465; doi:10.3390/biomedicines10123100)
Supplement: Supplementary file 1 [file biomedicines-10-03100-s001.zip › biomedicines-2049955-supplementary.pdf]

biomedicines

MDPI

Supplementary Figures

Immunohistochemical Detection of Estrogen Receptor-Beta (ERβ) with PPZ0506 Antibody in Murine Tissue: From Pitfalls to Optimization

Sarah K. Schröder, Carmen G. Tag, Jan C. Kessel, Per Antonson and Ralf Weiskirchen

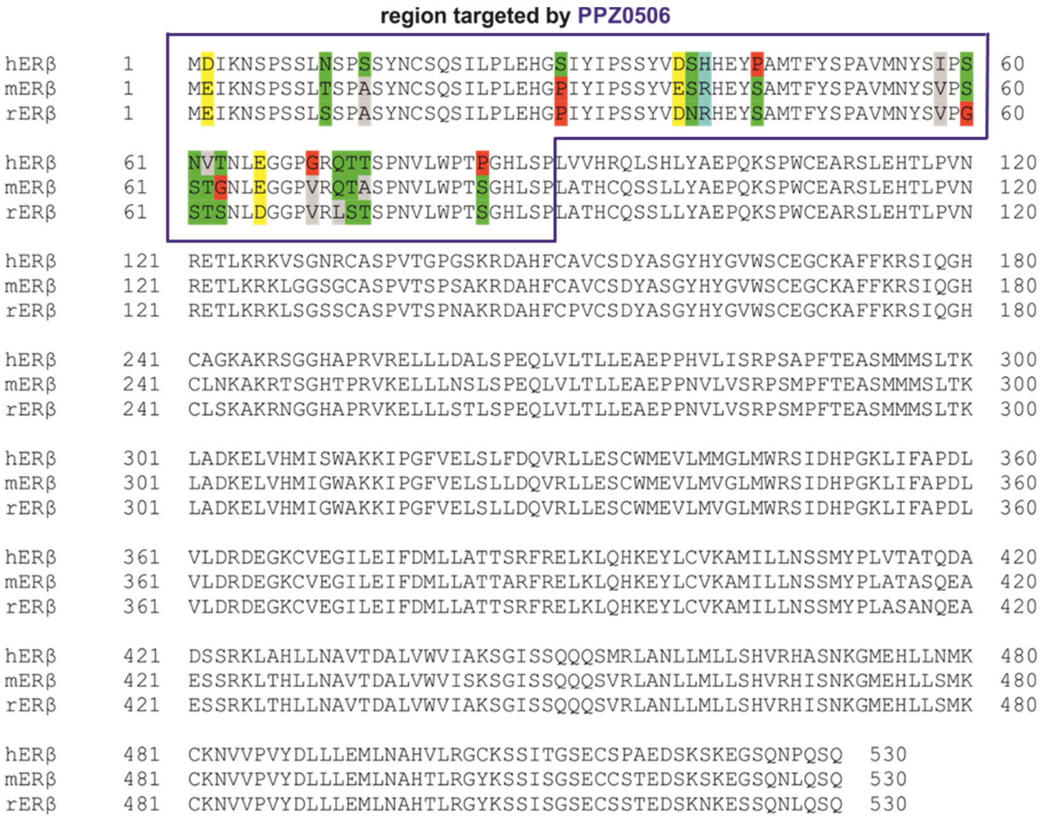

Protein sequence identity	Percentage identity to entire hERβ	Percentage identity to hERβ (2-88 AA)
mERβ	88.68%	83.91%
rERβ	88.30%	80.46%

Figure S1. Alignment of human and rodent estrogen receptor beta (ERβ) protein sequences. The entire protein sequences of mERβ and rERβ were aligned to hERβ using BLAST software. The *N*-terminal region containing the epitope recognized by the PPZ0506 antibody in hESR2 (2-88 amino acids (AA)) is labeled. The colors show the properties of the respective amino acid groups, as negatively charged at pH 7.0 (yellow), polar (green), non-polar (grey), affecting structure of the peptide (red), and positively charged at pH 7.0 (turquoise) in the antibody binding region. In addition, the percentages of identity of rodent ERβ to human ERβ (hERβ) are displayed.

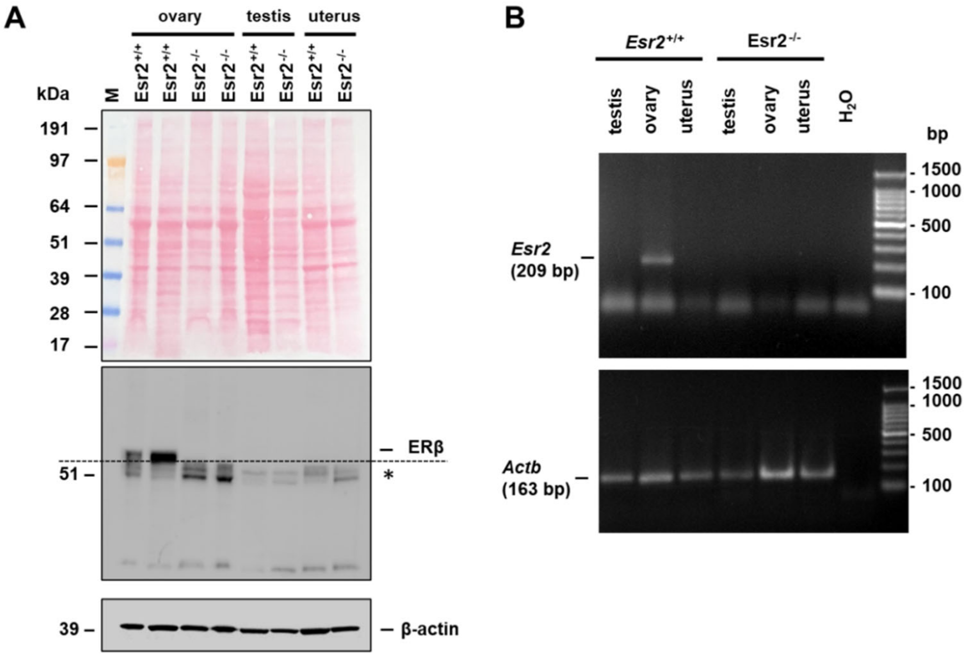

Figure S2. Estrogen receptor beta (ERβ) expression in reproductive tissues of *Esr2*$^{+/+}$ and *Esr2*$^{-/-}$ animals. Tissues were collected and processed for protein or mRNA analysis as described in Material and Methods. (**A**) ERβ protein expression was analyzed by western blot analysis in ovarian, uterine and testis tissue extracts using monoclonal PPZ0506 antibody. Ponceau S stain was used to confirm the successful protein transfer and re-probing with β-actin served to document equal protein loading in each lane. ERβ protein could only be detected in *Esr2*$^{+/+}$ ovaries but not in *Esr2*$^{-/-}$. The asterisk (*) indicates non-specific bands, proven by knockout animals where entire *Esr2* has been removed. (**B**) In line, RT-PCR amplified a 206-bp amplicon for *Esr2* in *Esr2*$^{+/+}$ ovary that was absent in all other tissues analyzed. Amplification of a 163-bp *Actb* fragment served as a control to document integrity of cDNA used as template.

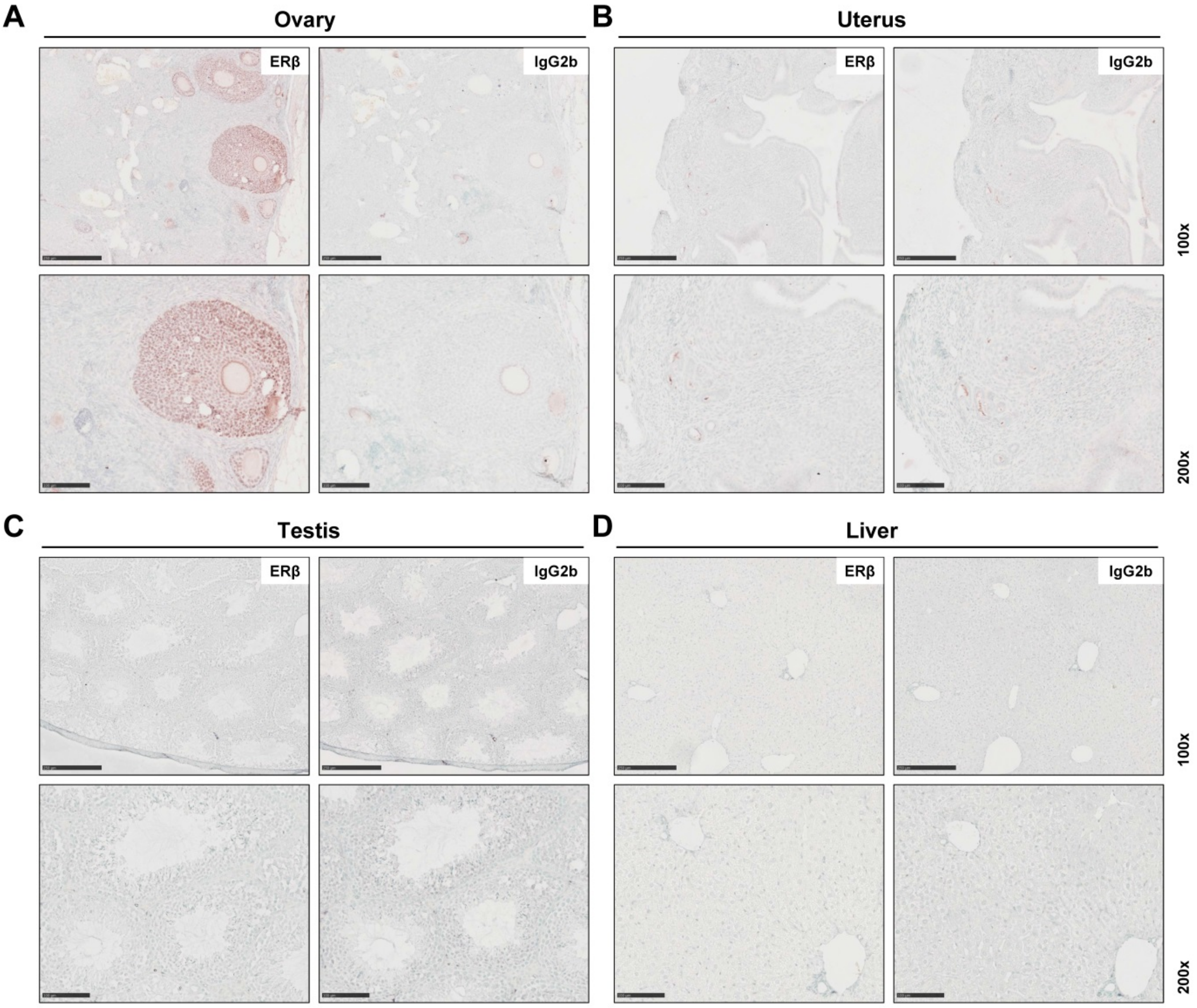

Figure S3. Estrogen receptor beta (ERβ) protein expression in different tissues. The expression of ERβ protein was studied in murine ovarian (**A**), uterine (**B**), testis (**C**) and liver tissue (**D**) with monoclonal PPZ0506 (1:6000) antibody or with isotype-specific IgG_{2b} negative control. Staining was performed using the optimized protocol and includes a HIER step as described in Materials and Methods. Please note that only in ovarian tissue (**A**) ERβ-positive cells were observed. Scale bars: 250 µm (100x) or 100 µm (200x).

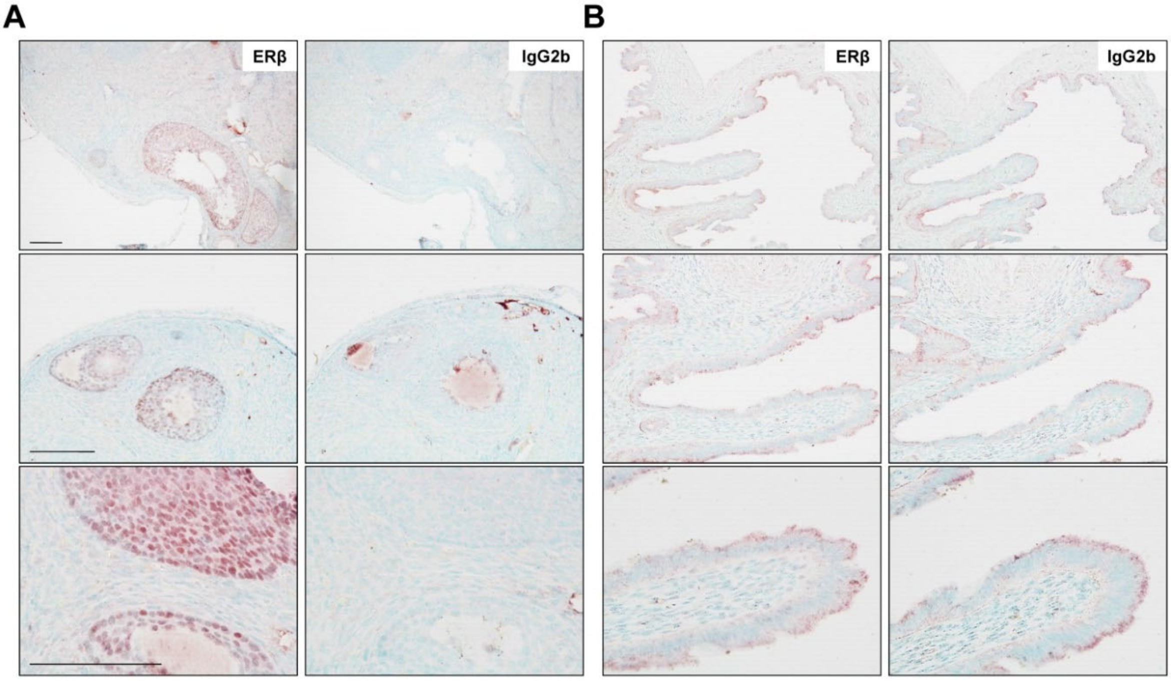

Figure S4. Mouse monoclonal IgG_{2b} was used as a negative control for PPZ0506 antibody. Murine ovaries (**A**) or uterine tissue (**B**) were processed as described in Material and Methods using the optimized protocol including HIER. Slices were incubated either with PPZ0506 (diluted 1:6000) or with IgG_{2b} as a negative control (same concentration as primary antibody). Please note that ovaries, incubated with IgG_{2b} do not show any specific nuclear staining for estrogen receptor beta (ERβ) in the granulosa cells. In uterine tissue, there is no specific staining and slight off-target signals are only visible. Scale bars 100 μm.
